# Health-based homophily in public housing developments

**DOI:** 10.1186/s12889-023-15146-4

**Published:** 2023-02-03

**Authors:** Brenda Heaton, Neha Gondal

**Affiliations:** 1 Department of Health Policy & Health Services Research, Boston University Henry M Goldman School of Dental Medicine, Boston, USA; 2 Department of Sociology and Faculty of Computing & Data Sciences, Boston University, Boston, USA; 3 Department of Epidemiology, Boston University School of Public Health, Boston, USA

**Keywords:** Ego-Centric Networks, Health Homophily, Health Interventions, Public Housing Developments, Multilevel Models

## Abstract

Public housing residents in the United States face disproportionately high risks for disease, presenting an urgent need for interventions. Evidence suggests interventions leveraging social networks can be successful when relationships are homophilous, as this leads to pooling of risk behaviors among interconnected alters. Yet, we know little about networks of public housing residents. To assess the feasibility of network-based interventions, we investigate the incidence of health-based homophily in public housing developments in Boston, Massachusetts. Employing multilevel models (HLM), we find that respondents report their own health characteristics to be similar to their network partners on oral health, weight, and consumption of sugar-sweetened beverages and foods. We discuss the implications of our findings for health-based interventions in low-income communities.

**Supplementary Information:**

The online version contains supplementary material available at 10.1186/s12889-023-15146-4.

## Introduction

Approximately one million households in the United States live in public housing developments (PHD) - a long-standing governmental model for housing low-income persons in urban areas. Residents of PHDs are typically of low socioeconomic status and, often, racial and ethnic minorities [1]. The processes propelling individuals into public housing are highly correlated with disease risk yielding socially and geographically distinct populations with some of the highest burdens of chronic disease [2]. A recent study in Boston, Massachusetts, for example, revealed that residence within family designated PHDs (homes allocated for low-income families) was associated with two or more emergency department visits in the previous year, high rates of exposure to smokers within households, and no dental visits in the last five years [3].

These disproportionately high burdens of risk have created an urgent need for interventions aimed at mitigating the behaviors and exposures that produce adverse health outcomes in these disadvantaged populations [e.g., 4–5]. Although individuals or entire communities have typically been the target of such interventions, evidence suggests that meso-level interventions that account for the social networks within which people are embedded can also be successful [6–9]. Social networks are collections of relationships interconnecting actors, and interventions targeting networks focus on clusters of linked persons. The success of such interventions depends on structural properties of networks (i.e., attributes of the network such as density and centrality); as well as the composition of networks, including homophily (i.e., the tendency for people who share characteristics and behaviors to form relationships with one another) [10, 11].

Homophily induces homogeneity in social networks so that attributes underlying the formation of homophilous ties are found to cluster in groups of connected persons. In addition to demographic attributes such as race and gender, homophily also characterizes health-related behaviors and outcomes. Research shows, for example, that social networks are homophilous on a variety of adverse health outcomes such as excessive alcohol consumption, smoking, and suicidal ideation as well as health-promoting behaviors including exercise, contraceptive use, undergoing mammography, and smoking cessation [6, 12–17]. Homogeneity produced through homophily in social networks limits exposure because less healthy individuals have fewer relatively healthy persons in their social networks and vice versa. Consequently, less healthy individuals have a lower likelihood of being exposed to the habits of their healthier counterparts as well as to health innovations adopted by such individuals [18].

These constraints have implications for interventions aimed at the prevention, cessation, and moderation of unhealthy behaviors. Interventions targeting unhealthy individuals may not be very successful if their networks are largely composed of actors facing similar risks and exhibiting comparable behaviors. The presence of such connections (or alters) in networks is likely to generate countervailing influences and pressures. Research on smoking cessation, for example, demonstrates that long-term success is generally negatively associated with having a partner who smokes as well as high proportions of smokers in close social networks [19]. Moreover, limits on exposure imply that innovations are unlikely to spread beyond the targeted individual and are, consequently, likely to be cost prohibitive. Research shows, for example, that discrepancy in exposures linked to homophily in networks can produce divergent rates of diffusion of practices and ideas among healthy and unhealthy groups [20]. Evidence in support of homophily and the consequent pooling of risk behaviors within social networks suggests that interventions focused on clusters of interconnected persons could provide a feasible alternative to individual- or community-based programs when networks are homophilous on health behaviors [6, 7, 21, 22]. In this vein, Christakis and Fowler [23] find smoking among adults to be clustered within networks and, significantly, cessation to also follow a similar pattern where groups of interconnected people are likely to quit smoking together.

The successful deployment of network-based interventions, thus, depends on the prevalence of health-based homophily in populations of interest. Yet, with few exceptions [e.g., 6, 24–25], research on the incidence of this form of homophily in the social networks of adults is scarce and tends to be centered on electronic media [e.g., 18, 26–27]. Even more problematically, despite the well-established association between low socioeconomic and racial minority status and chronic disease [28–30], little is known about the distribution of health outcomes and behaviors in the social networks of socioeconomically disadvantaged adults [31]. This lacuna is especially acute in the case of oral health and allied behaviors such as consumption of sugar-sweetened foods and beverages. Oral health status and access to care is characterized by significant sociodemographic disparities in the United States. Specifically, racial/ethnic minorities and those living in poverty are significantly less likely to have dental insurance and to receive preventive and restorative dental services [32]. Compared to those with higher socioeconomic status, these groups are significantly more likely to have untreated dental decay, experience dental pain, and greater tooth loss [33]. There is also growing recognition that dental diseases, such as caries and periodontal disease, are closely linked to other chronic health conditions like obesity and diabetes and to risk of stroke and cardiovascular disease [34, 35]. In light of persistent disparities in oral health, investigation of homophily on oral health in disadvantaged groups has the potential to inform much needed intervention strategies that can address multiple chronic disease outcomes.

Public housing developments are an appropriate site for this research for several reasons. Most significantly, axes of disadvantage tend to be profoundly concentrated within these communities. Government subsidized housing in the United States generally houses the very or extremely poor population and tends to be concentrated in racially segregated and low-income urban neighborhoods. Nine out of ten households residing in public housing in 2021 made less than 50% of the area median income, and more than three-fourths made less than 30% of that threshold [1]. The average household income of public housing residents in 2021 was $15,045, approximately one fifth of the national average. Two-thirds of public housing residents comprise racial or ethnic minorities. A quarter of all residents and nearly two-fifths of household heads also suffer from disabilities. Public housing is also distinct because disadvantage tends to be more concentrated among those living within this context than among those living in high poverty neighborhoods, in general, in the United States. Approximately 50% of those living in high-poverty neighborhoods own their own homes, for example, and a quarter make between $40,000 and $85,000 [36]. Finally, investigation of health disparities and feasibility of network-based interventions in public housing is necessary because public housing residents tend to have worse general and oral health outcomes than other Americans in comparable socioeconomic circumstances [2, 37, 38]. Indeed, the health of public housing residents has been described as being the worst of any population in the United States [39].

Our goal in this paper is to contribute to the small but growing body of literature investigating the prevalence of homophily on health-related outcomes and behaviors among public housing residents. Our data come from housing developments managed by the Boston Housing Authority. Public housing in Boston shares features with developments located across the United States. Additionally, Boston has been a ‘majority minority’ city for at least the last two decades, meaning that racial/ethnic minorities make up more than half of the city’s residents. Similar to many other urban areas in the United States, minorities in Boston tend to be densely populated in neighborhoods of the city where public housing developments are located, leading to the creation of a distinct social geography. Thus, although we focus on the Boston area, our results have some general applicability to other similarly positioned urban contexts in the United States.

## Data and methods

The data comprise egocentric social networks of residents of two public housing developments managed by the Boston Housing Authority (BHA). Both housing developments are designated for families, are of similar size (400–500 units), have similar distributions of self-identified race/ethnicity, and at least 30% of households with at least one child who was aged six or younger. The developments are located approximately 1.5 miles apart from each other. BHA provided the research team with a housing list, which served as the sampling frame from which households were selected for targeted recruitment. Caregivers aged 18–65 years, who were primarily responsible for providing care for a child aged 0–6 years, spoke either English or Spanish, and resided in one of the two selected public housing developments were targeted for participation. All eligible households were approached for participation. Subsequently, their ties were considered eligible to participate if they lived in any Boston PHD. Recruitment and enrollment took place between July 2016 and November 2016. The response rate was 55%. All interviews were conducted in-person by a trained interviewer, and the interviews ran fifty-one minutes long, on average. Written informed consent was obtained from all participants. A copy of the signed and dated consent form was left with the participant and the original was kept on file by the study team. We did not formally assess literacy; however, the regulatory elements of informed consent were reviewed by the interviewer with the study participants to ensure comprehension, and participants were read the consent form when necessary. Participants were provided with a $35 gift card to a local grocery or Target store as compensation for their time. The study protocol was approved by the Boston University Medical Campus Institutional Review Board and informed consent was obtained from all participants. All data procedures were carried out in accordance with relevant ethical guidelines and regulations associated with the declaration of Helsinki.

Social network data were gathered via ‘name generators,’ a means to gather data based on asking respondents for names of network members or alters with whom they have a particular type of relationship, followed by information on the relationship, as well as characteristics of alters. Name generators are generally considered to be the gold standard for collecting egocentric network data [40]. There is little consensus, however, on the best name generators for collecting such data [41]. Perry et al. [41] argue that choice of questions should be guided by theory, reflecting the social processes implicated in the setting. One popular approach is to construct questions that focus on the ‘functional’ characteristics of alters or the ‘content of exchange’ based on the goals of the research question. For example, if the research pertains to disease transmission in a population of drug users, it is appropriate to seek out needle-sharing networks. As our interest is in health and dietary consumption behaviors and outcomes, we asked respondents questions about meal sharing and grocery shopping ties. This is appropriate for our research because respondents are more likely to observe food and beverage consumption and purchasing behaviors in these contexts. Exchange-based name generators also tend to be less prone to variability in interpretation, leading to higher validity and reliability [42]. At the same time, focusing only on functionally specific questions can be limiting, so the inclusion of more general questions such as ‘with whom ego discusses important matters’ and ‘regularly interacts with’ captures a wider range of ties. The ‘discussing important matters’ question has been used in other surveys including the General Social Survey.

Based on this guidance, we used four name generators to elicit names of alters with whom respondents (egos) had (1) discussed important matters, (2) shared meals, (3) jointly done grocery shopping, and (4) regularly interacted in the PHD. The questions used to gather these data are available in a related file linked to this submission. We refer to these name generators as ‘types of ties’ in the following sections. There were no limits to the number of alters a respondent could nominate. A total of 655 alters were named by 118 egos. The number of alters named ranged from 1 to 13 (mean = 7.1; median = 7). After eliciting names, egos were asked to report on their own and their alters’ demographics as well as health characteristics and behaviors. Comparable to most other studies that use egocentric data [42], information on alters in our sample is, thus, gathered from respondents.

Accordingly, it is possible for the data to have measurement errors on alter characteristics. Specifically, if egos are prone to projecting evaluations about their own behaviors onto their network members, we would expect estimates of homophily to be inflated upwards. However, there are two reasons why this is not as problematic as it appears. First, evidence suggests that ego’s reports on the types of alter attributes and behaviors we measure can be quite accurate [e.g., 43–47]. Green et al. [44], for example, find that while egos are generally reliable informants, they are more accurate in regard to demographic attributes and, importantly, also in regard to health-related information, including HIV status (and whether alters were receiving HIV-related health care), as well as pregnancy status. White and Watkins [46], likewise, find that discrepancies between ego and alter reports are lower for observable characteristics. In contrast, ego reports on alter *attitudes* are more likely to be prone to be influenced by ego’s own position [42]. In our case, four of the five health-related behaviors we model, namely, oral health, weight, and consumption of sugar-sweetened beverages and foods, are likely to be reasonably observable. Prior research also shows that people who suffer from dental problems are likely to talk about these issues with network ties [48]. The implication is that even potentially less observable characteristics, such as oral health, are likely to be known to network partners. To confirm, we conducted some analyses on egos in our dataset that were also named as alters by other respondents. We provide some details about this analysis after the description of the variables. Overall, our analysis is supportive of the findings from prior research.

Second, biases in ego’s reports of network members’ behaviors are likely related to ego and alter demographic attributes such as age, gender, and ethnicity [e.g., 49–50]. For example, younger respondents may be more prone to viewing their alters as healthy. By controlling for attributes of both egos and alters in our models, we account for this issue better than models based on single level regressions. It is worth noting that other well-known instruments, including the General Social Survey and the World Values Survey, also gather data on alters from egos [51].

The relationship between ego’s reports of alter’s health (dependent variable) and ego’s own self-reported health (key independent variable) is tested using multilevel (hierarchical) binary logistic regression. We draw on the work of Snijders et al. [52] who demonstrate the utility of multilevel modeling for personal network data using two-level models – networks nested within individuals. The relations identified by each ego are likely to be interdependent and therefore should not be treated as independent observations in a standard regression. Consequently, if our interest is in ego reports of health-based homophily, we cannot treat the reported health of one alter choice (e.g., parent) as independent from the second (e.g., friend) for the same individual. To test our assumptions of interdependence, we calculated intraclass correlation coefficients (ICC) for our dependent variables, which reveals how the variance of the dependent variable is distributed across levels in a multilevel model. A high ICC suggests that, in general, the health behaviors of an ego’s alters are similar and, hence, for modeling purposes, likely interdependent. We find that four of the five ICCs are approximately 40% while the fifth – weight perceptions – is a little over 30%. This demonstrates that roughly one-third to two-fifths of the total variability in alters’ health and sugar consumption patterns is attributable to their connectivity to ego. Substantively, clustering within networks means that social relationships are relevant to assessments of health outcomes of public housing residents. Accordingly, multilevel models are appropriate.

We create five dependent variables, which occur at the alter-level, by binarizing ego reports of their alters’ health characteristics. These are: (1) alter’s oral health, (2) alter’s overall health, (3) alter’s weight, (4) alter’s consumption of sugar-sweetened beverages, and (5) alter’s consumption of sugar-sweetened foods. The first two variables were measured in five categories as ‘excellent’, ‘very good’, ‘good’, ‘fair’, and ‘poor’. The last two variables were also measured using a Likert scale as ‘rarely or never’, ‘at least once a week but not every day’, ‘once a day’, ‘twice a day’, and ‘more than three times a day.’ In both sets of variables, we combine the first three categories and the latter two categories to create a dichotomous variable where ‘0’ indicates better health or lower consumption levels. This coding decision is based on levels that have been previously associated with increased risk of deleterious health outcomes [e.g., 53–55]. For the final variable, egos were asked for their assessment of their own and alters’ weight levels in categories, which were ‘overweight’, ‘underweight’, and ‘about the right weight.’ We pooled the second two categories, which were coded as ‘0’. We conducted several sensitivity analyses by coding these variables in different ways. The outcomes of these tests are discussed briefly at the end of the Results section. Our analysis comparing ego’s self-reports with those of them named as alters by other respondents shows a considerable degree of consistency between the two measures. Specifically, we find that ego’s reports of alters matched ego’s self-reports of the dependent variables described above in a majority of the cases, ranging from 56% for consumption of sugar-sweetened foods to 72% for overall health.

Our independent variables occur at the alter and ego levels. At the alter level, we include a categorical variable indicating type of network tie (discussion alters, meal sharing alters, grocery shopping alters, and other public housing alters). We included this variable because we are interested in investigating if particular types of relationships are more homophilous than others. We do this by running models with interaction effects between type of tie and homophily. We also coded this variable into a fifth category of ‘multiplex’ ties – those individuals who were named in more than one category (e.g., discussion networks and meal sharing). Generally, multiplex ties are considered to be stronger than ties that pertain to a single type of relationship. Models with interaction effects test if multiplex ties are more prone to homophily than others. Accordingly, we set multiplex ties to be the omitted variable in the models. Changes to the omitted category did not produce meaningfully different results.

At the ego-level, our primary independent variable is the ego’s self-assessment of their own health and consumption patterns, coded the same way as the alter variables. In addition, we considered both ego and alter demographic variables as covariates in the model. Specifically, we include age as scaled and centered (continuous), race/ethnicity (Black, Non-Hispanic; Black, Hispanic; White, Non-Hispanic; White, Hispanic; Other), and education (Less than GED/High School, GED/High School Diploma, More than GED/High School). We also test if residence of alters in public, private, or other housing has an impact on health outcomes. Given that our primary independent variable occurs at the level of egos, we treat all variables other than the intercept as fixed. The structure of the model we employ is shown in Eq. 1 where γ is the average intercept, α coefficients are for alter-level, and β coefficients are for ego-level variables. U represents the variance of the intercept at the ego-level. All analyses were conducted using R [56].

1 $$\log it({P_{ijk}}) = {\gamma _0} + \sum\limits_{l = 1}^r {{\alpha _l}{x_{ijk}} + } \sum\limits_{m = 1}^s {{\beta _m}{y_{jk}} + } {U_{0j}}$$

## Results

Table 1 shows descriptive statistics for the dependent and control variables included in the models. Participants (egos) were predominantly female (87%), and approximately forty-one years of age. Nearly half (46%) reported Hispanic ethnicity and only 23% reported more than a high school education. Egos tended to report better overall health compared to oral health, with 70% indicating ‘Excellent, Very Good, Good’ overall health, and only 52% reporting oral health at the same level. Nearly half (49%) considered themselves as ‘overweight’, 65% reported daily consumption of sugar-sweetened beverages, and 31% reported daily consumption of sweet and sugary foods.

**Table 1:** Descriptive Statistics for Egos and Ego reports of Alters

Attributes	Ego (n = 118)	Alter (n = 655)
Average age in years (min, max)	40.7 (18, 80)	41.3 (16, 81)
Female (percent)	87.3	79.0
Hispanic Ethnicity (percent)	87.3	84.6
Race (percent)		
Non-Hispanic, Black or African American	17.8	17.3
Hispanic	45.8	48.9
White, Non-Hispanic	28.8	22.9
Multiracial/other	5.1	5.3
Don’t know/refused	2.5	5.7
Educational status (percent)		
Less than high school	37.3	30.0
High school or equivalent	39.8	43.8
More than high school	22.9	26.2
Residence prior to current public housing (percent)		
Public housing development	48.6	67.6
Private residence	22.9	27.9
Shelter	17.1	0.3
Outside of the US	4.8	0.0
Unspecified	6.7	4.2
**Self-Reported Health**		
Oral health (percent)		
Excellent, Very Good, or Good	51.7	73.7
Fair or Poor	48.3	26.3
General health (percent)		
Excellent, Very Good, or Good	70.4	75.5
Fair or Poor	29.6	24.5
Weight (percent)		
Underweight or Correct Weight	50.8	69.5
Overweight	49.1	30.4
Frequency of sugar-sweetened beverage consumption (percent)		
Rarely or never; at least once a week but not every day	35.0	38.6
Once, twice, or more than three times a day	64.9	61.4
Frequency of sugary food consumption (percent)		
Rarely or never; at least once a week but not every day	69.5	38.4
Once, twice, or more than three times a day	30.5	61.6

We fit three models for each outcome. First, we model the relationship between ego’s reports of their own health and their perception of alter health (e.g., oral health, overall health, weight, etc.) without any covariates. Our second model includes type of tie as a covariate, and our third model adds covariates at the level of the ego and alter.

Table 2 displays the model odds ratios for our three health status variables – oral health, overall health, and weight perception. Model coefficients and standard errors are available in Tables S1-S5 in the Supplementary Information file. Model 1 demonstrates a positive relationship between ego’s reports of their own oral health and that of her alters. Specifically, the odds of reporting an alter’s health as fair or poor (as opposed to excellent, very good, or good) are about 3.6 times as large for egos who consider their own oral health to be of similarly low quality than those who consider it to be of high quality. Alternatively stated, egos view their networks to be highly homophilous on oral health. Models 2 and 3 add a variety of control variables to the model equation. According to Model 2, a multiplex tie is no more likely to be perceived as being of poorer oral health than a tie belonging to any one category. Substantively this suggests that the type of tie connecting ego to alter has no effect on ego’s reports of alter’s oral health. Additionally, if we take multiplexity to be indicative of stronger ties, the results suggest that ego-alter closeness is also unrelated to homophily in oral health. Finally, the addition of tie-type has no effect on the relationship between ego and alter oral health.

**Table 2:** Multilevel Models showing odds ratios of ego and alter characteristics on Oral Health, Overall Health, and Weight Assessment

Variable	Oral Health			Overall Health			Weight Perception		
	Model 1	Model 2	Model 3	Model 1	Model 2	Model 3	Model 1	Model 2	Model 3
Intercept	0.13***	0.13***	0.06***	0.16***	0.15***	0.04***	0.26***	0.30***	0.11***
Oral Health Homophily	3.56***	3.53***	5.05***						
Overall Health Homophily				2.32*	2.36*	2.18			
Weight Perception Homophily							1.99*	2.08*	2.34**
*Network Categories*: Important Matters (Multiplex)		0.77	1.15		1.14	1.48		0.84	1.06
Shared Meals (Multiplex)		0.90	1.11		0.92	0.90		1. 21	1.62
Grocery Shopping (Multiplex)		0.84	1.30		0.45	0.52		0.54	0.73
Public Housing(Multiplex)		1.22	0.73		1.32	0.99		0.59*	0.52*
*Ego Demographics*: Female			0.81			2.01			1.06
Age			0.89			0.79			1.22
Non-Hispanic Black (Hispanic, Non-White)			0.58			0.14			0.40
Hispanic Black (Hispanic, Non-White)			1.36			0.26			0.75
Hispanic White (Hispanic, Non-White)			0.49			0.83			1.01
Other (Hispanic, Non-White)			1.65			0.35			1.30
No Formal Education (Higher Education)			2.08			2.18			0.79
GED/HS Diploma (Higher Education)			1.34			0.93			0.86
*Alter Demographics*: Female			1.31			1.30			1.65+
Age			2.66***			3.49***			1.05
Non-Hispanic Black (Hispanic, Non-White)			0.73			0.92			1.21
Hispanic Black (Hispanic, Non-White)			1.28			1.48			2.61
Hispanic White (Hispanic, Non-White)			0.88			1.04			1.95+
Other (Hispanic, Non-White)			0.94			0.80			0.84
No Formal Education (Higher Education)			2.59**			1.86+			0.70
GED/HS Diploma (Higher Education)			1.39			0.92			1.62+
Private Housing (Public Housing)			0.37***			0.69			0.92
Other Housing (Public Housing)			0.45			0.91			1.26
N (Alters)	631	631	569	652		585	653	652	586
N (Egos)	118	118	111	118		111	118	118	111

(Omitted categories in Parentheses)

Significance Levels: ***(0.001); **(0.01); *(0.05); +(0.01)

Model 3, which incorporates demographic variables for ego and alter, shows that older alters tend to be of poorer oral health. Compared to alters with higher levels of education, alters with no formal education are also perceived to be of poorer health. Finally, alters who live in private housing are considered to have superior oral health than those who live in public housing. Except for ego’s own oral health assessment, no other variables at this level have any bearing on alters’ oral health. This demonstrates that ego’s own attributes do not systematically affect their perception of alters’ oral health. The implication is that potential biases in ego’s reports on alter behaviors, if any, do not vary systematically by ego’s own characteristics.

The next set of results in Table 2 pertains to overall health assessments. Egos who view their own overall health negatively are likely to be connected to others whose health is also perceived to be poor. The odds of reporting an alter’s overall health to be of low quality are about 2.3 times as large for egos who consider themselves to be unhealthy than those who consider themselves to be in good health. The inclusion of type of tie in the regression equation has no effect on this relationship. Yet, the significance of the homophily variable (ego’s own health assessment) disappears when demographic variables are added to the model. The model also shows that alter age is related to ego’s perception of alters’ overall health with older alters being viewed as less healthy than younger ones. Likewise, less educated alters are reported to be of poorer overall health.

Evaluations of perceived weight status are also homophilous (Model 1). Unlike either of the previous health-related outcomes, type of relationship influences ego’s assessment of alter’s weight. Specifically, as compared to multiplex ties, other ties in public housing (with whom one interacts outside of home but does not discuss important matters, share meals, or does groceries) are less likely to be overweight. Ego characteristics have no bearing on alter weight assessments. However, female alters, those with lower levels of education such as a GED or High School diploma (as compared to those who have higher levels of education), and White Hispanics (as compared to Hispanic, non-white) alters are more likely to be reported as overweight. Interaction effects between type of tie and homophily did not produce statistically significant results for any of the variables modeled in Table 2.

Table 3 displays the model coefficients for our health behavior outcomes - sugar-sweetened beverages (SSB) and foods (SSF). Baseline Models for both outcomes demonstrate homophily on sugar consumption levels. The addition of type of tie to the equation leaves the relationship between ego and alter consumption levels nearly unchanged. Certain types of ties are more likely to be perceived as having higher levels of SSB or SSF consumption. In the case of beverages, network members with whom one shares meals or does groceries are reported as having higher levels of sugar consumption than members with whom one has a fuller, more multiplex relationship. With respect to food, network members with whom ego only does groceries or those who live in public housing are reported as higher-level consumers.

**Table 3:** Multilevel Models showing odds ratios of ego and alter characteristics on consumption of Sugar-Sweetened Beverages and Sugar-Sweetened Foods

Variable	Sugar Sweetened Beverages			Sugar Sweetened Foods		
	Model 1	Model 2	Model 3	Model 1	Model 2	Model 3
Intercept	0.89	0.67	1.22	1.30	0.94	2.23
Sugar-Sweetened Beverages Homophily	3.39***	3.56***	3.03*			
Sugar-Sweetened Foods Homophily				2.64*	2.77**	2.16+
*Network Categories*: Important Matters (Multiplex)		1.20	1.63		1.35	1.42
Shared Meals (Multiplex)		1.97*	2.29*		2.05*	2.51**
Grocery Shopping (Multiplex)		3.74**	4.31**		1.90	2.10+
Public Housing(Multiplex)		1.49	1.90*		1.84*	1.65+
*Ego Demographics*: Female			0.11**			0.24*
Age			1.02			0.80
Non-Hispanic Black (Hispanic, Non-White)			1.05			0.90
Hispanic Black (Hispanic, Non-White)			1.16			0.76
Hispanic White (Hispanic, Non-White)			0.48			0.78
Other (Hispanic, Non-White)			5.64+			0.83
No Formal Education (Higher Education)			0.98			0.51
GED/HS Diploma (Higher Education)			1.23			0.46
*Alter Demographics*: Female			1.34			1. 58+
Age			0.75*			0.91
Non-Hispanic Black (Hispanic, Non-White)			2.72+			2.23
Hispanic Black (Hispanic, Non-White)			3.97+			1.65
Hispanic White (Hispanic, Non-White)			2.72+			2.12
Other (Hispanic, Non-White)			0.70			1.72
No Formal Education (Higher Education)			3.56***			1.80*
GED/HS Diploma (Higher Education)			1.86*			1.58+
Private Housing (Public Housing)			1.88*			1.02
Other Housing (Public Housing)			0.68			0.93
N (Alters)	631	631	581	652		588
N (Egos)	118	118	110	118		111

(Omitted categories in Parentheses)

Significance Levels: ***(0.001); **(0.01); *(0.05); +(0.01)

Alters in the ‘important matters’ network, in contrast, are not reported as consuming elevated amounts of sugar-sweetened foods or beverages. Models with interaction effects between type of tie and ego consumption of SSB and SSF did not produce statistically significant results. Homophily of SSB as well as SSF persists in the final models adjusted for ego and alter demographic variables. In the case of beverages, older alters are less likely but less educated ones are more likely to be reported as high-level consumers. The odds of perceiving an alter’s consumption of SSB are about 3.6 times as large for those with no formal education than those with at least some higher levels of education. Alters with no formal education are also more likely to consume higher levels of sugar-sweetened foods. As compared to male egos, female egos report that their alters consume lower levels of SSB as well as SSF.

Results for three types of sensitivity analyses we conducted are shown in Tables S6-8 in the Supplementary Information file. Table S1 shows results for a multilevel logistic regression based on an alternative coding scheme from the one used in the results above. Specifically, for oral health, overall health, and consumption of sugar-sweetened foods and beverages, we combine the first two categories of the original variable to denote better health (coded as ‘0’) and the latter three categories as indicators of poor health (coded as ‘1’). For weight, we compare ‘overweight’ and ‘underweight’ to ‘about the right weight.’ We also ran single-level models, shown in Table S7, testing for homophily between ego behaviors and proportion of alters with similar behavior patterns [e.g., 6, 24–25]. Finally, results shown in Table S8 are based on treating ego and alter behaviors as continuous variables in a multilevel linear regression. The coefficients from these sensitivity analyses are in the same direction and substantively similar to the ones we discuss in the main body of the paper.

## Discussion and conclusion

The spatial clustering of poverty and disease risk among residents of public housing developments across the United States has produced an urgent need for mitigating those risk factors. Recent research suggests that interventions leveraging homophilous social networks can yield successful outcomes. Yet, we have scant research on the prevalence of homophily in low-income disadvantaged communities. We contribute to the scholarship in this area by investigating ego networks of public housing residents in Boston, Massachusetts. We find that networks are homophilous on subjective assessments of oral health, weight, and consumption of sugar-sweetened beverages as well as foods. Homophily is strongest for oral health followed by sugar-sweetened beverages.

Our approach in treating ego-networks as multilevel data also offers an additional insight. We show that homophily persists after accounting not only for relevant ego attributes, as others have shown, but also alter demographics. Without multilevel models, findings of homophilous relationships between ego and ‘proportion of alters’ with the behavior (this aggregation is necessary for single-level models) could arise because egos tend to befriend alters with specific characteristics that are correlated with health outcomes. For example, our data show that older alters tend to be of worse oral and overall health. If certain types of egos in poor health tend to have mostly older persons as their alters, their networks would appear to be more homophilous than may be the case empirically.

The results also have implications for intervention strategies. First, Valente [9] argues that two types of programs are especially effective in inducing behavioral change: (1) providers, who attempt to persuade clients to change their behaviors in clinical settings and (2) outreach workers, who go door-to-door in affected communities to contact clients in their own homes. The success of these approaches hinges on interpersonal communication through sources perceived to be credible, knowledgeable, and trustworthy. This type of contact also helps to reinforce mass media messages, which may not be taken as seriously. Despite these advantages, such strategies are labor-intensive rendering them limited in their capacity to target large audiences. Moreover, prior research [e.g., 19; 21–23] shows that interpersonal intervention techniques may be less effective in the absence of adequate social support and in the presence of countervailing influences from close personal contacts. Our findings show that social networks comprising close personal ties of public housing residents tend to be homophilous on a variety of health outcomes. This finding can be leveraged in order to expand the reach and success of interpersonal intervention strategies by shifting the target from the individual to their social network. That is, rather than convincing a single individual, providers and outreach workers attempt to persuade a person and their close personal ties to simultaneously change their habits and behaviors. Focusing on social networks would not only stem influences that run counter to the intervention but also generate positive influence from close personal ties who are themselves invested in producing behavioral change. In addition to improving the chances of success, this would also be a more effective strategy for utilizing limited resources by lowering program costs.

Practically, targeting social networks would involve supplementing resource-intensive door-to-door programs with initiatives that entail asking a smaller set of individuals for names of their close confidantes with similar behavioral profiles and targeting some proportion of those persons. Likewise, asking patients or clients for names of close ties that share behaviors or exposures and including them in intervention programs could increase the likelihood of long-term success. These approaches are especially valuable in the context of public housing developments, our chosen research site, because such locations are typically characterized by clear community boundaries, which make for homophilous social networks (as we show), and population characteristics that are of intervention interest at the community level (i.e., designated for families, a presence of caregivers with young children). More generally, in homophilous contexts, focusing on networks of interconnected persons with similar risk profiles ought to be considered as a complementary approach for producing sustained change in behaviors.

Our findings also suggest that interventions may work to mitigate a wide range of health outcomes [57]. Consumption of sugar-sweetened beverages, for example, has received heightened attention as a key modifiable risk factor for poor oral health across all age groups particularly in relation to the development of dental decay [58]. Further, dietary sugars generally, and sugar-sweetened beverage consumption specifically, have been strongly linked to the development of obesity [59]. Thus, policies aimed at mitigating sugar-sweetened beverage consumption can be expected to have positive effects on several disease risk factors. Third, our use of multilevel models also provides some additional insights into interventions based on the type or context of ties. Daw et al. [31], for example, show that levels of homophily on health behaviors like smoking and drinking among adolescents varies by type of relationship suggesting that some types of ties make better targets for intervention. To test for this in our data, we ran models with interaction effects between types of ties and homophily. We found no evidence that particular types of relationships are more homophilous than others. This finding suggests that intervention programs can target a variety of relational contexts such as localized neighborhoods, family, and shared transportation for grocery shopping.

There are some limitations in our study. First, as discussed in the Data and Methods section, data on alter characteristics are sourced from egos. Far from novel, this is standard practice in ego-centric network analysis. Moreover, as argued above, focusing on largely observable health conditions and controlling for demographic attributes in our models should mitigate potential biases in reports. We also find evidence in favor of consistency between ego self-reports and others’ reports for the subset of respondents that were also named as alters in our sample. Issues of bias in ego reports on alters may also not be problematic if our concern is primarily in assessing potential for health-based interventions. Homophily facilitates behavioral adoption and diffusion because individuals are more likely to be influenced by those who are similar to them. Studies on health-based diffusion using online platforms show, however, that it is ego’s *perceptions* of alter behavior (rather than empirical behavior) that is important in this process. Network partners in online platforms typically see only limited information about their alters like their gender, age, and BMI [18]. More specifically, egos have no direct communication with their alters to adjudicate the veracity of that information. Rather, they simply believe their alters to be similar to them. Indeed, Centola finds that subjects who observed adoption by someone who they understood to be similar to them along gender and BMI were more likely to adopt behaviors themselves. Centola explains that this is likely because signals from homophilous others are perceived as more relevant. Wang et al. [60], likewise, show that participants in online health discussion groups viewed information as having greater credibility and were more likely to act on the received advice if they perceived their alters to be homophilous. Thus, to the extent egos’ evaluations of alters’ behaviors are reasonably accurate and if perceptions of homophily are the relevant parameter for behavioral adoption, then ego’s reports on alter are a valid instrument to locate homophily and assess its implications for health-based interventions.

A second potential limitation of our work is that our data are sourced from public housing developments in one city. Although residents of these developments share characteristics in common with inhabitants of similar neighborhoods in other cities, it is possible that the specific characteristics of Boston as a majority minority city in the Northeast lend less generalizability to our findings. Gathering data from disparate locations or replicating the study in other urban centers with public housing would improve our understanding of homophily on health outcomes and behaviors in housing developments. Third, our data are limited by the types of questions we use to elicit social networks. It is possible that including a larger number of name generator questions would yield more varied and widespread network connections. Another benefit of more comprehensive network data could be a higher degree of sociometric connectivity, conducive to testing the effects of network structural properties such as centrality. Finally, our study investigates conditions that make for successful health-based interventions, but does not adopt an interventionist approach. Future practice-based research should investigate the effects of health-based homophily on intervention outcomes in low-income housing. It is worth noting, however, that while prior research suggests that homophily is important to adoption of behaviors in social networks [18], there is little scientific research focused on leveraging the effects of homophily on health-based interventions. The little research that does exist tends to investigate the spread of information on online platforms [61]. Indeed, we could not find any studies that draws on homophily in social networks to implement health-focused interventions in a client-based or other offline context.

In light of this paucity in research, our findings that, much like other behaviors such as smoking, oral health and consumption of sugar-sweetened beverages and foods is a socially clustered phenomenon in low-income neighborhoods, are especially significant. Although not shown here, we also find social networks to be homophilous on gender, education, and race/ethnicity. This multiplicity or layering of homophily generates deeply homogenous social networks that are highly conducive to the diffusion of behaviors and information [20]^62^. The existence of such overlapping forms of homophily in public housing networks suggests that appropriately designed interventions targeting social networks could be promising for mitigating some of the burdens of chronic disease in this vulnerable population.

## Electronic supplementary material

Below is the link to the electronic supplementary material.

Supplementary Material 1

## Data Availability

The datasets generated and/or analyzed during the current study are not publicly available due to confidentiality reasons. Data can be made available upon reasonable request from Brenda Heaton (brenda9@bu.edu).
