# Supplementary material for: Health-based homophily in public housing developments
Source: BMC Public Health. 2023 Feb 3;23:238. doi: 10.1186/s12889-023-15146-4 (PMC9896682; doi:10.1186/s12889-023-15146-4)
Supplement: Supplementary file 1 — Supplementary Material 1 [file 12889_2023_15146_MOESM1_ESM.docx]

**Supplementary Information**

**Table S1: Multilevel Models showing unadjusted coefficients and standard errors of ego and alter characteristics
on Oral Health**

| **Variable** | **Oral Health** | | | | | |
| --- | --- | --- | --- | --- | --- | --- |
|  | **Model 1** | **Std. Err.** | **Model 2** | **Std. Err.** | **Model 3** | **Std. Err.** |
| Intercept | -2.07 | 0.25 | -2.02 | 0.28 | -2.86 | 0.97 |
| Oral Health Homophily | 1.27 | 0.34 | 1.26 | 0.34 | 1.62 | 0.47 |
| *Network Categories*: Important Matters (Multiplex) |  |  | -0.26 | 0.25 | 0.14 | 0.33 |
| Shared Meals (Multiplex) |  |  | -0.11 | 0.33 | 0.1 | 0.37 |
| Grocery Shopping (Multiplex) |  |  | -0.17 | 0.41 | 0.26 | 0.45 |
| Public Housing (Multiplex) |  |  | 0.2 | 0.26 | -0.32 | 0.3 |
| *Ego Demographics:* Female |  |  |  |  | -0.21 | 0.75 |
| Age |  |  |  |  | -0.12 | 0.25 |
| Non-Hispanic Black (Hispanic, Non-White) |  |  |  |  | -0.55 | 1.12 |
| Hispanic Black (Hispanic, Non-White) |  |  |  |  | 0.31 | 0.91 |
| Hispanic White (Hispanic, Non-White) |  |  |  |  | -0.72 | 0.64 |
| Other (Hispanic, Non-White) |  |  |  |  | 0.5 | 0.91 |
| No Formal Education (Higher Education) |  |  |  |  | 0.73 | 0.64 |
| GED/HS Diploma (Higher Education) |  |  |  |  | 0.29 | 0.63 |
| *Alter Demographics*: Female |  |  |  |  | 0.27 | 0.28 |
| Age |  |  |  |  | 0.98 | 0.13 |
| Non-Hispanic Black (Hispanic, Non-White) |  |  |  |  | -0.31 | 0.59 |
| Hispanic Black (Hispanic, Non-White) |  |  |  |  | 0.25 | 0.72 |
| Hispanic White (Hispanic, Non-White) |  |  |  |  | -0.13 | 0.54 |
| Other (Hispanic, Non-White) |  |  |  |  | -0.06 | 0.51 |
| No Formal Education (Higher Education) |  |  |  |  | 0.95 | 0.34 |
| GED/HS Diploma (Higher Education) |  |  |  |  | 0.33 | 0.31 |
| Private Housing (Public Housing) |  |  |  |  | -1 | 0.29 |
| Other Housing (Public Housing) |  |  |  |  | -0.8 | 0.6 |
| Fit Statistic | 631 |  | 631 |  | 569 |  |
| N (Alters) | 118 |  | 118 |  | 111 |  |

**Table S2: Multilevel Models showing unadjusted coefficients and standard errors of ego and alter characteristics
on Overall Health**

| **Variable** | **Overall Health** | | | | | |
| --- | --- | --- | --- | --- | --- | --- |
|  | **Model 1** | **Std. Err.** | **Model 2** | **Std. Err.** | **Model 3** | **Std. Err.** |
| Intercept | -1.84 | 0.21 | -1.89 | 0.25 | -3.22 | 1.05 |
| Overall Health Homophily | 0.84 | 0.37 | 0.86 | 0.37 | 0.78 | 0.56 |
| *Network Categories*: Important Matters (Multiplex) |  |  | 0.13 | 0.25 | 0.39 | 0.33 |
| Shared Meals (Multiplex) |  |  | -0.08 | 0.33 | -0.11 | 0.38 |
| Grocery Shopping (Multiplex) |  |  | -0.8 | 0.47 | -0.65 | 0.52 |
| Public Housing (Multiplex) |  |  | 0.28 | 0.25 | -0.01 | 0.29 |
| *Ego Demographics:* Female |  |  |  |  | 0.71 | 0.84 |
| Age |  |  |  |  | -0.24 | 0.29 |
| Non-Hispanic Black (Hispanic, Non-White) |  |  |  |  | -1.99 | 1.28 |
| Hispanic Black (Hispanic, Non-White) |  |  |  |  | -1.36 | 1.1 |
| Hispanic White (Hispanic, Non-White) |  |  |  |  | -0.19 | 0.63 |
| Other (Hispanic, Non-White) |  |  |  |  | -1.05 | 0.97 |
| No Formal Education (Higher Education) |  |  |  |  | 0.78 | 0.66 |
| GED/HS Diploma (Higher Education) |  |  |  |  | -0.07 | 0.66 |
| *Alter Demographics*: Female |  |  |  |  | 0.26 | 0.29 |
| Age |  |  |  |  | 1.25 | 0.14 |
| Non-Hispanic Black (Hispanic, Non-White) |  |  |  |  | -0.08 | 0.69 |
| Hispanic Black (Hispanic, Non-White) |  |  |  |  | 0.39 | 0.79 |
| Hispanic White (Hispanic, Non-White) |  |  |  |  | 0.04 | 0.53 |
| Other (Hispanic, Non-White) |  |  |  |  | -0.22 | 0.48 |
| No Formal Education (Higher Education) |  |  |  |  | 0.62 | 0.34 |
| GED/HS Diploma (Higher Education) |  |  |  |  | -0.08 | 0.34 |
| Private Housing (Public Housing) |  |  |  |  | -0.37 | 0.29 |
| Other Housing (Public Housing) |  |  |  |  | -0.09 | 0.61 |
| Fit Statistic | 652 |  |  |  | 585 |  |
| N (Alters) | 118 |  |  |  | 111 |  |

**Table S3: Multilevel Models showing unadjusted coefficients and standard errors of ego and alter characteristics on
Weight Perceptions**

| **Variable** | **Weight Perception** | | | | | |
| --- | --- | --- | --- | --- | --- | --- |
|  | **Model 1** | **Std. Err.** | **Model 2** | **Std. Err.** | **Model 3** | **Std. Err.** |
| Intercept | -1.34 | 0.21 | -1.21 | 0.24 | -2.17 | 0.71 |
| Weight Perception Homophily | 0.69 | 0.29 | 0.73 | 0.3 | 0.85 | 0.31 |
| *Network Categories*: Important Matters (Multiplex) |  |  | -0.17 | 0.23 | 0.06 | 0.28 |
| Shared Meals (Multiplex) |  |  | 0.19 | 0.31 | 0.48 | 0.34 |
| Grocery Shopping (Multiplex) |  |  | -0.62 | 0.41 | -0.31 | 0.43 |
| Public Housing (Multiplex) |  |  | -0.52 | 0.25 | -0.66 | 0.27 |
| *Ego Demographics:* Female |  |  |  |  | 0.06 | 0.51 |
| Age |  |  |  |  | 0.2 | 0.18 |
| Non-Hispanic Black (Hispanic, Non-White) |  |  |  |  | -0.92 | 0.82 |
| Hispanic Black (Hispanic, Non-White) |  |  |  |  | -0.29 | 0.66 |
| Hispanic White (Hispanic, Non-White) |  |  |  |  | 0.01 | 0.43 |
| Other (Hispanic, Non-White) |  |  |  |  | 0.26 | 0.66 |
| No Formal Education (Higher Education) |  |  |  |  | -0.23 | 0.44 |
| GED/HS Diploma (Higher Education) |  |  |  |  | -0.15 | 0.43 |
| *Alter Demographics*: Female |  |  |  |  | 0.5 | 0.26 |
| Age |  |  |  |  | 0.05 | 0.11 |
| Non-Hispanic Black (Hispanic, Non-White) |  |  |  |  | 0.19 | 0.54 |
| Hispanic Black (Hispanic, Non-White) |  |  |  |  | 0.96 | 0.59 |
| Hispanic White (Hispanic, Non-White) |  |  |  |  | 0.67 | 0.4 |
| Other (Hispanic, Non-White) |  |  |  |  | -0.18 | 0.44 |
| No Formal Education (Higher Education) |  |  |  |  | -0.35 | 0.29 |
| GED/HS Diploma (Higher Education) |  |  |  |  | 0.48 | 0.28 |
| Private Housing (Public Housing) |  |  |  |  | -0.08 | 0.25 |
| Other Housing (Public Housing) |  |  |  |  | 0.23 | 0.5 |
| Fit Statistic | 653 |  | 652 |  | 586 |  |
| N (Alters) | 118 |  | 118 |  | 111 |  |

**Table S4: Multilevel Models showing unadjusted coefficients and standard errors of ego and alter characteristics
on Sugar Sweetened Beverages**

| **Variable** | **Sugar Sweetened Beverages** | | | | | |
| --- | --- | --- | --- | --- | --- | --- |
|  | **Model 1** | **Std. Err.** | **Model 2** | **Std. Err.** | **Model 3** | **Std. Err.** |
| Intercept | -0.12 | 0.27 | -0.4 | 0.31 | 0.2 | 0.97 |
| Sugar Sweetened Beverages Homophily | 1.22 | 0.35 | 1.27 | 0.36 | 1.11 | 0.48 |
| *Network Categories*: Important Matters (Multiplex) |  |  | 0.18 | 0.24 | 0.49 | 0.3 |
| Shared Meals (Multiplex) |  |  | 0.68 | 0.32 | 0.83 | 0.35 |
| Grocery Shopping (Multiplex) |  |  | 1.32 | 0.44 | 1.46 | 0.49 |
| Public Housing (Multiplex) |  |  | 0.4 | 0.25 | 0.64 | 0.29 |
| *Ego Demographics:* Female |  |  |  |  | -2.19 | 0.76 |
| Age |  |  |  |  | 0.02 | 0.23 |
| Non-Hispanic Black (Hispanic, Non-White) |  |  |  |  | 0.05 | 0.97 |
| Hispanic Black (Hispanic, Non-White) |  |  |  |  | 0.15 | 0.88 |
| Hispanic White (Hispanic, Non-White) |  |  |  |  | -0.74 | 0.64 |
| Other (Hispanic, Non-White) |  |  |  |  | 1.73 | 0.91 |
| No Formal Education (Higher Education) |  |  |  |  | -0.02 | 0.59 |
| GED/HS Diploma (Higher Education) |  |  |  |  | 0.21 | 0.56 |
| *Alter Demographics*: Female |  |  |  |  | 0.29 | 0.26 |
| Age |  |  |  |  | -0.29 | 0.12 |
| Non-Hispanic Black (Hispanic, Non-White) |  |  |  |  | 1 | 0.56 |
| Hispanic Black (Hispanic, Non-White) |  |  |  |  | 1.38 | 0.73 |
| Hispanic White (Hispanic, Non-White) |  |  |  |  | 1 | 0.51 |
| Other (Hispanic, Non-White) |  |  |  |  | -0.36 | 0.45 |
| No Formal Education (Higher Education) |  |  |  |  | 1.27 | 0.31 |
| GED/HS Diploma (Higher Education) |  |  |  |  | 0.62 | 0.28 |
| Private Housing (Public Housing) |  |  |  |  | 0.63 | 0.25 |
| Other Housing (Public Housing) |  |  |  |  | -0.38 | 0.51 |
| Fit Statistic |  |  |  |  |  |  |
| N (Alters) | 631 |  | 631 |  | 581 |  |
| N (Egos) | 118 |  | 118 |  | 110 |  |

**Table S5: Multilevel Models showing unadjusted coefficients and standard errors of ego and alter characteristics
on Sugar Sweetened Foods**

| **Variable** | **Sugar Sweetened Foods** | | | | | |
| --- | --- | --- | --- | --- | --- | --- |
|  | **Model 1** | **Std. Err.** | **Model 2** | **Std. Err.** | **Model 3** | **Std. Err.** |
| Intercept | 0.26 | 0.2 | -0.06 | 0.24 | 0.8 | 0.86 |
| Sugar Sweetened Foods Homophily | 0.97 | 0.37 | 1.02 | 0.38 | 0.77 | 0.45 |
| *Network Categories*: Important Matters (Multiplex) |  |  | 0.3 | 0.24 | 0.35 | 0.28 |
| Shared Meals (Multiplex) |  |  | 0.72 | 0.32 | 0.92 | 0.34 |
| Grocery Shopping (Multiplex) |  |  | 0.64 | 0.4 | 0.74 | 0.43 |
| Public Housing (Multiplex) |  |  | 0.61 | 0.25 | 0.5 | 0.28 |
| *Ego Demographics:* Female |  |  |  |  | -1.42 | 0.67 |
| Age |  |  |  |  | -0.22 | 0.21 |
| Non-Hispanic Black (Hispanic, Non-White) |  |  |  |  | -0.1 | 0.9 |
| Hispanic Black (Hispanic, Non-White) |  |  |  |  | -0.27 | 0.77 |
| Hispanic White (Hispanic, Non-White) |  |  |  |  | -0.25 | 0.55 |
| Other (Hispanic, Non-White) |  |  |  |  | -0.19 | 0.77 |
| No Formal Education (Higher Education) |  |  |  |  | -0.67 | 0.53 |
| GED/HS Diploma (Higher Education) |  |  |  |  | -0.78 | 0.5 |
| *Alter Demographics*: Female |  |  |  |  | 0.46 | 0.25 |
| Age |  |  |  |  | -0.09 | 0.11 |
| Non-Hispanic Black (Hispanic, Non-White) |  |  |  |  | 0.8 | 0.53 |
| Hispanic Black (Hispanic, Non-White) |  |  |  |  | 0.5 | 0.66 |
| Hispanic White (Hispanic, Non-White) |  |  |  |  | 0.75 | 0.47 |
| Other (Hispanic, Non-White) |  |  |  |  | 0.54 | 0.42 |
| No Formal Education (Higher Education) |  |  |  |  | 0.59 | 0.3 |
| GED/HS Diploma (Higher Education) |  |  |  |  | 0.46 | 0.27 |
| Private Housing (Public Housing) |  |  |  |  | 0.02 | 0.24 |
| Other Housing (Public Housing) |  |  |  |  | -0.07 | 0.49 |
| Fit Statistic |  |  |  |  |  |  |
| N (Alters) | 652 |  |  |  | 588 |  |
| N (Egos) | 118 |  |  |  | 111 |  |

Table S6: Sensitivity analyses showing unadjusted coefficients and standard errors from multilevel logistic regressions for the homophily variable for Models 1 and 3 as configured in the tables above and the main manuscript. Coefficients of control variables and covariates are suppressed. Dependent and key independent variables for Oral and Overall health are coded dichotomously: 0 = ‘excellent’ and ‘very good’ and 1 = ‘good’, ‘fair’, and ‘poor.’ Dependent and key independent variables for SSB and SSF are coded dichotomously: 0 = ‘rarely or never’ and ‘at least once a week but not every day’ and 1 = ‘once a day’, ‘twice a day’, ‘more than three times a day.’ The dependent and key independent variable for weight is coded dichotomously: 0 = ‘overweight’ and ‘underweight’ and 1 = ‘about the right weight.’

|  | **Oral** | | **Overall** | | **Weight** | | **SSB** | | **SSF** | |
| --- | --- | --- | --- | --- | --- | --- | --- | --- | --- | --- |
|  | **Coeff** | **SE** | **Coeff** | **SE** | **Coeff** | **SE** | **Coeff** | **SE** | **Coeff** | **SE** |
| **Model 1** | 1.46 | 0.69 | 2.34 | 0.70 | 0.67 | 0.27 | 1.22 | 0.35 | 0.97 | 0.37 |
| **Model 3** | 1.76 | 0.91 | 2.45 | 0.89 | 0.70 | 0.28 | 1.11 | 0.48 | 0.77 | 0.45 |

Table S7: Sensitivity analyses showing unadjusted coefficients and standard errors from logistic regressions for the homophily variable for Models 1 and 3 as configured in the tables above and the main manuscript. Coefficients of control variables and covariates are suppressed. Dependent variables are ego behavior/weight/consumption levels and key independent variable is proportion of alters with the same behavior/weight/consumption level.

|  | **Oral** | | **Overall** | | **Weight** | | **SSB** | | **SSF** | |
| --- | --- | --- | --- | --- | --- | --- | --- | --- | --- | --- |
|  | **Coeff** | **SE** | **Coeff** | **SE** | **Coeff** | **SE** | **Coeff** | **SE** | **Coeff** | **SE** |
| **Model 1** | 2.30 | 0.72 | 1.47 | 0.68 | 1.4977 | 0.66 | 1.75 | 0.56 | 1.63 | 0.63 |
| **Model 3** | 2.41 | 0.76 | 1.03 | 0.80 | 1.47 | 0.69 | 1.93 | 0.70 | 1.19069 | 0.71 |

Table S8: Sensitivity analyses showing unadjusted coefficients and standard errors from multilevel linear regressions for the homophily variable for Models 1 and 3 as configured in the tables above and the main manuscript. Coefficients of control variables and covariates are suppressed. Dependent variables are alter behavior/weight/consumption levels treated as linear variables and the key independent variables are ego behavior/weight/consumption levels also treated as linear variables.

|  | **Oral** | | **Overall** | | **SSB** | | **SSF** | |
| --- | --- | --- | --- | --- | --- | --- | --- | --- |
|  | **Coeff** | **SE** | **Coeff** | **SE** | **Coeff** | **SE** | **Coeff** | **SE** |
| **Model 1** | 0.79 | 0.71 | 0.46 | 0.18 | 0.27 | 0.06 | 0.21 | 0.07 |
| **Model 3** | 0.76 | 0.69 | 0.20 | 0.23 | 0.17 | 0.07 | 0.10 | 0.08 |
